# Reducing Cd Uptake by Wheat Through Rhizosphere Soil N-C Cycling and Bacterial Community Modulation by Urease-Producing Bacteria and Organo-Fe Hydroxide Coprecipitates

**DOI:** 10.3390/microorganisms13061412

**Published:** 2025-06-17

**Authors:** Junqing Zhang, Shuangjiao Tang, Hao Wei, Lunguang Yao, Zhaojin Chen, Hui Han, Mingfei Ji, Jianjun Yang

**Affiliations:** 1 Henan Key Laboratory of Ecological Security for Water Source Region of Mid-Line of South-to-North Diversion Project, Collaborative Innovation of Water Security for the Water Source Region of the Mid-Line of the South-to-North Diversion Project of Henan Province, Nanyang Normal University, Nanyang 473061, China; jq_zhang2018@163.com (J.Z.); 18836758365@163.com (S.T.); 18336085389@163.com (H.W.); lunguangyao@nynu.edu.cn (L.Y.); zhaojin_chen@163.com (Z.C.); jimfdy@gmail.com (M.J.); 2 State Key Laboratory of Efficient Utilization of Arable Land in China, Institute of Agricultural Resources and Regional Planning, Chinese Academy of Agricultural Sciences, Beijing 100081, China

**Keywords:** cadmium immobilization, urease-producing bacteria, organo-Fe hydroxide coprecipitates, wheat, rhizosphere microbiome

## Abstract

The bioavailability of heavy metals is profoundly influenced by their interactions with active soil components (microorganisms, organic matter, and iron minerals). However, the effects of urease-producing bacteria combined with organo-Fe hydroxide coprecipitates (OFCs) on Cd accumulation in wheat, as well as the mechanisms underlying these effects, remain unclear. In this study, pot experiments integrated with high-throughput sequencing were employed to investigate the impacts of the urease-producing bacterial strain TJ6, ferrihydrite (Fh), and OFCs on Cd enrichment in wheat grains, alongside the underlying soil–microbial mechanisms. The results demonstrate that the strain TJ6-Fh/OFC consortium significantly (*p* < 0.05) reduced (50.1–66.7%) the bioavailable Cd content in rhizosphere soil while increasing residual Cd fractions, thereby decreasing (77.4%) Cd accumulation in grains. The combined amendments elevated rhizosphere pH (7.35), iron oxide content, and electrical conductivity while reducing (14.5–21.1%) dissolved organic carbon levels. These changes enhanced soil-colloid-mediated Cd immobilization and reduced Cd mobility. Notably, the NH_4_^+^ content and NH_4_^+^/NO_3_^−^ ratio were significantly (*p* < 0.05) increased, attributed to the ureolytic activity of TJ6, which concurrently alkalinized the soil and inhibited Cd uptake via competitive ion channel interactions. Furthermore, the relative abundance of functional bacterial taxa (*Proteobacteria*, *Gemmatimonadota*, *Enterobacter*, *Rhodanobacter*, *Massilia*, *Nocardioides*, and *Arthrobacter*) was markedly increased in the rhizosphere soil. These microbes exhibited enhanced abilities to produce extracellular polymeric substances, induce phosphate precipitation, facilitate biosorption, and promote nutrient (C/N) cycling, synergizing with the amendments to immobilize Cd. This study for the first time analyzed the effect and soil science mechanism of urease-producing bacteria combined with OFCs in blocking wheat’s absorption of Cd. Moreover, this study provides foundational insights and a practical framework for the remediation of Cd-contaminated wheat fields through microbial–organic–mineral collaborative strategies.

## 1. Introduction

Cadmium (Cd) is recognized as one of the most concerning contaminants in soil due to its high toxicity, mobility, and bioaccumulation potential [1]. Statistical data indicate that 7.0% of monitored sites in China exceed Cd contamination thresholds, ranking it among the top inorganic pollutants [2]. Cd contamination in agricultural soils poses a severe threat to global food security and ecosystem health. In China, widespread Cd pollution has been documented in key farming regions. Concentrations in critical areas, such as the carbonate-rich soils of southwest China, average 0.571 mg kg^−1^—nearly double that of non-carbonate regions—and those in the sewage-irrigated fields of Hebei Province reach 3.88 mg kg^−1^ [3,4]. This pervasive contamination translates to tangible hazards, including carcinogenic risks (CRs > 10^−6^) and elevated hazard quotients (HQs) via dietary intake, particularly for children. Moreover, Cd disrupts plant physiology by inhibiting root elongation, impairing nutrient uptake, and inducing oxidative stress, thereby reducing wheat biomass and grain yield [5,6]. Consequently, developing efficient, cost-effective, and eco-friendly remediation technologies for Cd-contaminated soils holds significant scientific and practical value. Conventional remediation approaches face notable limitations. Physical methods such as soil replacement effectively reduce Cd concentrations in the short term but require substantial financial and labor investments [7]. Chemical passivators can fix heavy metals in the soil through adsorption, reducing their mobility. However, they are costly and may cause secondary pollution [8]. In contrast, bioremediation has emerged as a promising alternative due to its environmental compatibility, cost efficiency, and reduced secondary pollution risks [9].

Cd bioavailability in the soil–plant system is governed by its speciation dynamics at the root–soil interface. In the rhizosphere, Cd exists primarily as exchangeable, carbonate-bound, Fe/Mn-oxide-bound, organic-matter-bound, and residual fractions, with the exchangeable and carbonate-bound fractions exhibiting high bioavailability [10]. Redox fluctuations induced by water management (e.g., flooding vs. drainage) drive cyclic dissolution–precipitation of iron oxides, thereby regulating Cd release or sequestration. Soil pH further modulates these processes: acidic conditions (pH < 6.5) increase Cd desorption from mineral surfaces via H^+^ competition, whereas alkaline environments (pH > 7.5) favor Cd coprecipitation with carbonates [11]. Additionally, anions (e.g., Cl^−^ and SO_4_^2−^) form soluble complexes (e.g., CdCl^+^) that enhance Cd mobility by 20–40%, and organic amendments (e.g., hydrochar) reduce bioavailability through humic acid complexation and microbial immobilization [12]. These physicochemical interactions underscore the need for integrated strategies targeting rhizosphere Cd speciation.

Soil bacteria, ubiquitously distributed in soil environments, interact with Cd through diverse mechanisms, such as biosorption and biotransformation [13]. The main mechanisms by which functional bacteria reduce the bioavailability of Cd in the soil include the following: (1) surface adsorption and ion exchange. The functional groups on the surface of microbial cells (such as carboxyl, hydroxyl, amino, and phosphate groups) combine with heavy metal ions through electrostatic interaction, coordination bonds, or ion exchange [14]. (2) Intracellular accumulation and chelation. Heavy metals form stable complexes with intracellularly synthesized metal sulfoproteins, plant chelators, or glutathione and are then stored in vacuoles or the cytoplasm [15]. (3) Bioprecipitation. Proteolytic bacteria decompose urea to produce NH_3_ and CO_3_^2−^, increasing the environmental pH and promoting the formation of carbonate precipitates of heavy metals [16]. (4) The interaction between biofilms and EPSs. The EPSs secreted by microorganisms or the formed biofilms provide adsorption sites and precipitation substrates for heavy metals [17]. The mechanisms by which functional bacteria prevent crops from absorbing heavy metals is a complex process involving microbial–soil–plant interactions. This mechanism reduces the migration of heavy metals to crops by altering the environmental behavior of heavy metals (such as morphology, toxicity, and mobility) or regulating the absorption and transport pathways of plants [18]. On the one hand, bacterial inoculants or inhibitors reduce the available content of heavy metals through chelation, precipitation, redox transformation, or competitive ion exchange. On the other hand, bacteria colonizing the surfaces of plant roots form biofilms or secrete EPSs, forming a “filter layer” around the roots to prevent heavy metal ions from directly contacting the root system. Bacteria induce plants to form a “plasmoplast-epiplasmic” transport barrier, causing heavy metals to be retained in the cell walls or vacuoles of the root cells and reducing their transport to the xylem. In addition, bacteria can induce plants to synthesize metal sulfoproteins or plant chelators, enhancing the intracellular chelation ability of heavy metals [19]. Organic carbon, a critical soil component, not only serves as a carbon and energy source for microbial communities but also modifies Cd speciation by forming complexes or chelates with Cd ions [20]. Iron oxides, common mineral constituents in soils, exhibit large specific surface areas and high surface charges, endowing them with robust Cd adsorption capacities. Bacteria utilize organic carbon as a nutrient source for growth and reproduction, while their metabolic byproducts influence organic carbon decomposition and transformation [21]. Extracellular polymeric substances secreted by bacteria further facilitate the formation and stabilization of iron oxides, which in turn provide colonization sites and nutrient reservoirs for bacterial communities [22]. Organic carbon modulates the Cd adsorption efficiency of iron oxides through surface interactions, while iron oxide adsorption of organic carbon alters the latter’s ability to interact with Cd [23]. These interdependent relationships underscore the necessity of elucidating the mechanisms underlying tripartite-interaction-mediated Cd immobilization. Such insights are pivotal for unraveling Cd’s environmental behavior in soils and advancing the development of efficient bioremediation technologies for Cd-contaminated agricultural systems.

Notably, iron oxides in soil frequently combine with organic carbon through electrostatic interactions, ligand exchange, hydrophobic interactions, hydrogen bonding, and cation bridging to form organo-Fe hydroxide coprecipitates (OFCs) [24]. Within the ternary system of iron oxides, organic carbon, and heavy metals, organic carbon exerts dual and opposing effects on the adsorption and immobilization of heavy metals on iron oxides. On the one hand, organic carbon can chelate heavy metals, facilitating the adsorption of metal ions onto iron oxide surfaces [25]. On the other hand, organic carbon may compete with metal ions for adsorption sites on iron oxides, thereby suppressing their capacity for heavy metal retention [26]. Xia et al. [27] demonstrated that elevated dissolved organic carbon (DOC) loading reduced Cr adsorption on OFCs. In our preliminary study, the incorporation of corn-straw-derived DOC similarly diminished Cd adsorption on ferrihydrite (Fh). However, the urease-producing bacterial strain TJ6 counteracted this limitation by oxidizing the FeO on OFC surfaces to Fe_2_O_3_ via its intrinsic oxidative activity, thereby enhancing the Cd adsorption capacity of OFCs [28]. Furthermore, strain TJ6 immobilized Cd^2+^ through ion exchange involving hydrogen ions (H^+^) at primary and secondary amide functional groups on its cell surface. Nevertheless, the combined effects of strain TJ6 and OFCs on Cd bioavailability in soil remain poorly understood, particularly regarding Cd uptake by wheat, warranting further investigation.

In this study, strain TJ6, Fh, OFCs, TJ6+Fh, and TJ6+OFCs were added to the soil, and wheat pot experiments were set up to investigate (1) the effects of different passivator treatments on the contents of available Cd, DOC, and NH_4_^+^ in the rhizosphere soil; (2) the influence of different passivator treatments on Cd uptake in wheat; and (3) the effects of different passivator treatments on the bacterial community structure of the rhizosphere soil of wheat. These research results provide technical approaches for the safe production of wheat fields contaminated with heavy metals.

## 2. Materials and Methods

### 2.1. Strain TJ6, Fh, and OFCs

The heavy-metal-immobilizing bacterium *Enterobacter bugandensis* TJ6 (MK836418) was isolated from Cd-contaminated soil, demonstrating urease secretion capability for inducing carbonate precipitation of heavy metals [29]. Fh (Fe_5_HO_8_·4H_2_O, purity 95%) was synthesized by dissolving 1.4 g of FeCl_3_ in 50 mL of deionized water and adjusting the pH to 7.0–8.0 with 1 mol L^−1^ HNO_3_ to form dark reddish-brown precipitates, followed by 24 h of agitation at 200 rpm, centrifugation at 5000 rpm for 10 min, two deionized water washes, and 48 h freeze-drying before 100-mesh sieving. OFCs with an Fe/C molar ratio of 5 were prepared by first dissolving 60.8 g of FeCl_3_·6H_2_O in 1 L of deionized water, mixing 50 g of corn stalk powder with 500 mL of water at 4 °C (180 rpm, 24 h) to produce the DOC solution, combining 90 mL of FeCl_3_·6H_2_O with 30 mL of the DOC solution, and diluting the mixture to 900 mL with 10 mmol L^−1^ NaCl (pH 4.5). The reaction proceeded at 25 °C (180 rpm, 24 h), and finally, precipitates were collected via centrifugation (5000 rpm, 10 min), followed by two washes and 48 h of lyophilization [26].

### 2.2. Wheat Pot Experiment

A total of 100 kg of sieved Cd-contaminated soil (Fluvo-aquic soil) was weighed. Soil properties: 3.54 mg kg^−1^ Cd, pH 7.22, 25.46 g kg^−1^ organic matter, 0.47 g kg^−1^ available P, and 33.1 cmol(+) kg^−1^ cation-exchange capacity. To enhance soil fertility and meet wheat growth requirements, 100 g of urea, 50 g of KH_2_PO_4_, and 45 g of KCl were added and thoroughly mixed before being portioned into pots (5 kg per pot). The experiment comprised six treatment groups with three replicates each: (1) untreated control (CK), (2) 0.5% Fh amendment, (3) 0.5% OFC amendment, (4) inoculation strain TJ6 (TJ6), (5) combined 0.5% Fh and TJ6 inoculation (Fh+TJ6), and (6) combined 0.5% OFC and TJ6 inoculation (OFC+TJ6). Seeds of the wheat cultivar Jimai-22 were surface-sterilized by soaking them in 75% ethanol for 2 min, rinsed twice with deionized water, and placed on moist gauze for germination in a 25 °C incubator (48 h). Germinated seeds were evenly sown in pots and lightly covered with dry soil (20 seedlings retained per pot). Next, 25 g of OFC/Fh was uniformly mixed with 5 kg of naturally air-dried soil. The mixture was left to stand at 25 °C for 90 days while maintaining the soil moisture content at 30%. Subsequently, the wheat was planted. Strain TJ6 was activated in LB medium, expanded, and adjusted to OD_600_ = 1.0 (1.0 × 10^8^ CFU mL^−1^) with deionized water. At seedling emergence (soil moisture content at 40%), 15 mL of the bacterial suspension (per pot) was pipetted along the wheat roots. A second inoculation was performed at the jointing stage. The control groups (CK, Fh, OFC) received an equivalent volume of deionized water. Wheat was cultivated under standard conditions until maturity.

### 2.3. Effects of Different Treatments on Dry Weight and Cd Content of Wheat Grains

At wheat maturity and harvest, grains from each treatment were collected and oven-dried at 80 °C until constant weight was achieved. The wheat grains were ground into powder using a mortar, and 0.1 g of the sample was weighed into a 50 mL polypropylene crucible. For digestion, 4.5 mL of HNO_3_, 1.5 mL of HCl, and 2.0 mL of HClO_4_ were added, followed by hotplate digestion. After complete digestion, the solution was diluted to 10 mL with 1% nitric acid. The Cd concentration in the digestate was determined using inductively coupled plasma optical emission spectrometry (ICP-OES). National standard reference materials (GBW-08501 [30]) were used for quality control, and the results of the standard sample determination were all within the allowable error range.

### 2.4. Determination of Physicochemical Properties of Rhizosphere Soil

The distribution of different Cd fractions (exchangeable, carbonate-bound, Fe-Mn-oxide-bound, organic-matter-bound, and residual Cd) in rhizosphere soil was determined using the Tessier five-step sequential extraction method [31]. Briefly, solutions of various forms of Cd were extracted sequentially with 1 mol L^−1^ MgCl_2_, 1 mol L^−1^ NaAc, 0.04 mol L^−1^ HAC, and 0.02 mol L^−1^ HNO_3_. After adding the digestion solution (15 mL of HNO_3_, 10 mL of HF, 5 mL of HClO_4_) and performing electrothermal digestion, the content of Cd in the digestion solution was determined using ICP-OES. National standard reference materials (GBW-07402 [32]) were used for quality control, and the results of the standard sample determination were all within the allowable error range. Soil pH was measured at a soil-to-water ratio of 1:2.5 (*w*/*v*) using a pH meter. The organic matter content in rhizosphere soil was determined using the potassium dichromate–sulfuric acid titration method [33]. For soil electrical conductivity (EC) measurement, air-dried soil was placed in a 250 mL Erlenmeyer flask, mixed with 100 mL of deionized water, and shaken at 180 rpm for 30 min to ensure thorough mixing. After standing for 30 min, the supernatant was filtered into a 100 mL beaker, and the electrode was immersed to measure conductivity. The content of free Fe was determined using the sodium dithionite–citrate–bicarbonate (DCB) method [34]. Briefly, 1 g of soil sample was placed in a 50 mL centrifuge tube, mixed with 30 mL of extractant (a solution containing 0.27 mol L^−1^ sodium citrate and 0.11 mol L^−1^ sodium bicarbonate, adjusted to pH 7.3 with HCl), and heated in an 80 °C water bath for 15 min. Then, 0.5 g of sodium dithionite was added, and heating continued for another 15 min. After centrifugation at 4000 rpm for 10 min, the supernatant was transferred to a 250 mL volumetric flask, and the free iron content was measured using ICP-OES. NH_4_^+^-N and NO_3_^−^-N contents in the rhizosphere soils were measured using spectrophotometric methods [33].

### 2.5. Determination of TOC and DOC Content

The total organic carbon (TOC) content in the wheat rhizosphere soil was determined using the potassium dichromate oxidation–external heating method [35]. For the measurement of dissolved organic carbon (DOC) content, air-dried soil samples were mixed with 0.5 mol L^−1^ K_2_SO_4_ at a ratio of 1:5, shaken at 220 rpm for 1 h at 25 °C, and then centrifuged at 7000 rpm for 10 min to obtain the supernatant. The DOC content was measured using a total organic carbon analyzer (Shimadzu TOC-L CPH, Kyoto, Japan) [36].

### 2.6. Determination of Bacterial Community Structure in Rhizosphere Soil

Fresh rhizosphere soil samples (0.2 g) from different wheat treatments were collected, and total DNA was extracted using the E.Z.N.A.^®^ Soil DNA Kit (Omega Bio-tek, Norcross, GA, USA). The V3-V4 hypervariable regions of the 16S rRNA gene were amplified by PCR following the method described by Wang et al. [37], using the forward primer 338F (5′-ACTCCTACGGGAGGCAGCAG-3′) and the reverse primer 806R (5′-GGACTACHVGGGTWTCTAAT-3′). Sequencing and analysis were performed on the Illumina Nextseq2000 platform (Shanghai Majorbio Bio-pharm Technology Co., Ltd., Shanghai, China). Taxonomic classification of ASVs was conducted using the Naive Bayes classifier in Qiime2 based on the Silva 16S rRNA gene database (v138). Principal coordinate analysis (PCoA) is an unconstrained dimensionality reduction method. It enables the inter-group comparison of species diversity across different habitats or microbial communities, facilitating the exploration of similarities or dissimilarities in community composition among samples from different groups. The Bray–Curtis dissimilarity algorithm was employed to calculate pairwise distances between samples. This distance metric is calculated based on independent taxonomic units (such as OTUs) and does not incorporate phylogenetic relationships or association information among species. The significance of observed differences in community structure (*p* < 0.05) was assessed using PERMANOVA. Following beta-diversity comparisons of the overall community structure, differential species contributing to these differences were further identified (differential species analysis). PCoA statistical analyses and graphical visualizations were performed using R software (version 3.3.1). Redundancy analysis (RDA) was performed and visualized using the vegan package (version 2.4.3) in R. Response matrix: Bacterial community composition at the genus level (relative abundance data). Explanatory matrix: Environmental variables including EX-Cd, OM-Cd, Fe-Mn-Cd, CB-Cd, RES-Cd, TOC, DOC, pH, EC, NH_4_^+^-N, NO_3_^−^-N, and free Fe content. Data transformation: To address the characteristics of compositional data (containing numerous zeros), the response matrix (genus-level relative abundances) underwent Hellinger transformation. This reduces the influence of rare species and improves adherence to linear model assumptions. Explanatory variables (environmental factors) were standardized (centered and scaled to unit variance) to eliminate scale effects. Multicollinearity control: Prior to constructing the final explanatory matrix, multicollinearity among environmental predictors was assessed by calculating variance inflation factors (VIFs). Predictors with VIF values exceeding a threshold of 10 were sequentially removed. The final RDA model included only environmental variables with VIF < 10. The prediction of 16S rRNA gene functions was performed using PICRUSt2 (version 2.2.0).

### 2.7. Statistical Analysis

Data were analyzed using Excel 2019 and SPSS 26.0, and visualization and graphing were performed using Origin 2022. Mathematically processed results are presented in the form of M ± SE. Each of the three replicates (n = 3) in each treatment of this study is an independent sample, not three repeated tests of the same sample. Non-parametric alternatives and Tukey’s test (*p* < 0.05) were used to compare the treatment means. Microbial diversity data analysis was conducted on the Meiji Bio Cloud platform (https://cloud.majorbio.com).

## 3. Results

### 3.1. Dry Weight and Cd Content of Wheat Grain

**Figure 1:**
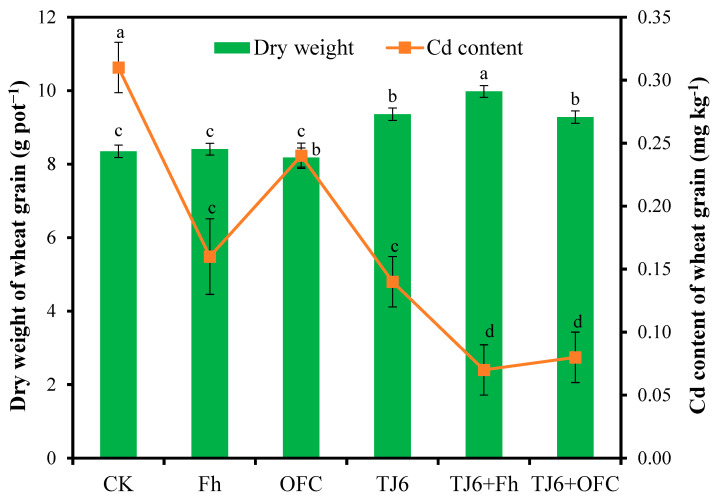
Effects of different treatments on dry weight and Cd content of wheat grains. Values are mean plus standard deviation (n = 3), and data with different lowercase letters are significantly different at *p* < 0.05 according to Tukey’s HSD test.

Compared to the CK treatment, Fh and OFCs had no significant (*p* < 0.05) effects on the dry weight of wheat grains, while TJ6, TJ6+Fh, and TJ6+OFCs significantly (*p* < 0.05) increased it by 12.1–19.5% (Figure 1), indicating that strain TJ6 was able to promote wheat growth. The Cd content in wheat grains in the CK treatment was 0.31 mg kg^−1^, while those in wheat grains treated with Fh, OFCs, and TJ6 were 0.16, 0.24, and 0.14 mg kg^−1^, respectively (Figure 1), suggesting that these passivators effectively inhibited Cd uptake by wheat grains. Notably, the Cd concentrations in wheat grains treated with TJ6+Fh and TJ6+OFCs were 0.07 mg kg^−1^ and 0.08 mg kg^−1^, which are below China’s food safety threshold (0.1 mg kg^−1^), demonstrating dual benefits, namely, environmental Cd immobilization and agricultural product safety.

### 3.2. Morphological Distribution of Cd in Wheat Rhizosphere Soil

**Figure 2:**
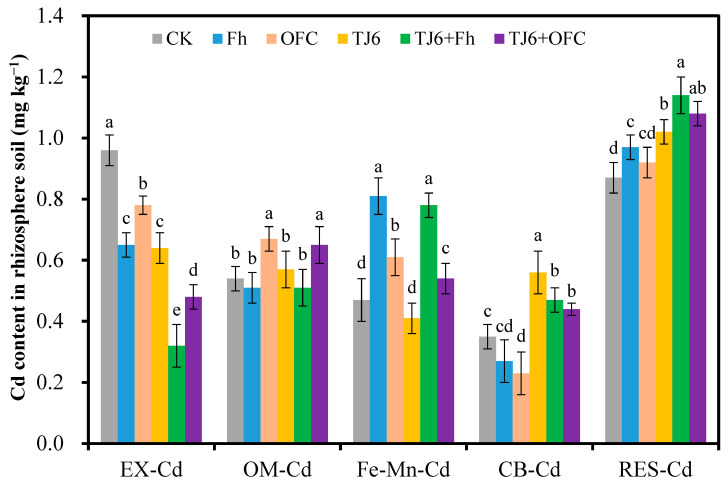
The effects of different treatments on the content of available Cd in wheat rhizosphere soil. The error bars represent ± standard errors (n = 3). The bars indicated by different letters for each Cd morphology are significantly different (*p* < 0.05) according to Tukey’s HSD test. EX-Cd: exchangeable Cd; OM-Cd: organic-matter-bound Cd; Fe-Mn-Cd: Fe-Mn-oxide-bound Cd; CB-Cd: carbonate-bound Cd; and RES-Cd: residual Cd.

Compared to the CK group, all passivators significantly (*p* < 0.05) reduced the EX-Cd content in wheat rhizosphere soil (Figure 2). Among them, the content of EX-Cd in the rhizosphere soil of the CK group was 0.96 mg kg^−1^. Fh and OFCs reduced the content of EX-Cd in the rhizosphere soil by 32.3% and 18.7%, respectively, while TJ6, TJ6+Fh, and TJ6+OFCs decreased the content of EX-Cd in the rhizosphere soil by 33.3%, 66.7%, and 50.1%, respectively. Fh and TJ6 exhibited higher Cd immobilization capacity than OFCs, while TJ6+Fh demonstrated the strongest Cd immobilization ability among all passivators. Compared to the CK group, OFCs and TJ6+OFC significantly (*p* < 0.05) increased the OM-Cd content in wheat rhizosphere soil by 20.1% and 24.3%, respectively, whereas Fh and TJ6+Fh notably increased the Fe-Mn-Cd content (65.9–72.3%) (Figure 2). Additionally, TJ6, TJ6+OFCs, and TJ6+Fh significantly (*p* < 0.05) elevated the CB-Cd content by 25.7%, 34.3%, and 60.2%, respectively. Furthermore, the application of TJ6 and Fh significantly (*p* < 0.05) increased the RES-Cd content, which may be attributed to the inherent properties of the passivators. In summary, the combination of TJ6 and Fh effectively reduced the bioavailable Cd content in wheat rhizosphere soil while increasing the levels of Fe-Mn-Cd, CB-Cd, and RES-Cd.

### 3.3. Effects of Different Treatments on Soil Properties

**Figure 3:**
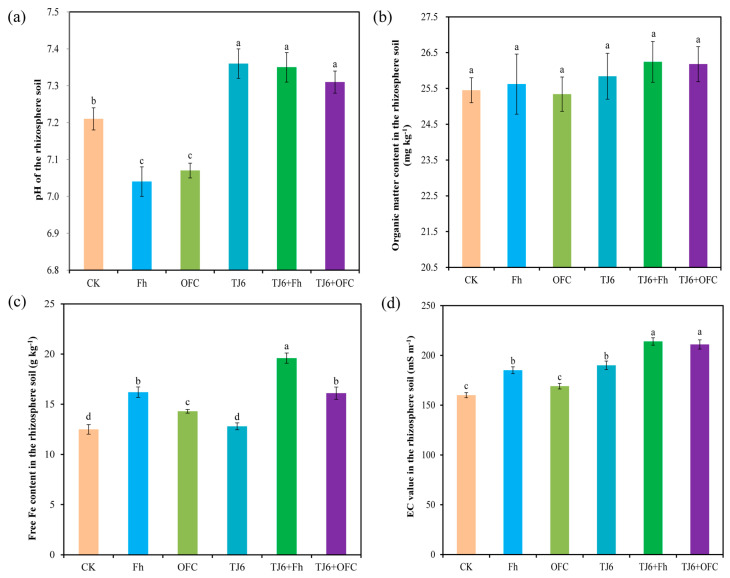
Effects of different treatments on pH (**a**), organic matter content (**b**), free Fe content (**c**), and EC content (**d**) of wheat rhizosphere soil. The error bars represent ± standard errors (n = 3). The bars indicated by different letters are significantly different (*p* < 0.05) according to Tukey’s HSD test.

In the control group, the pH of the rhizosphere soil was 7.24. Both Fh and OFCs significantly (*p* < 0.05) reduced the pH of the rhizosphere soil, while the inoculation of strain TJ6 increased it (Figure 3a). Notably, the addition of TJ6+Fh and TJ6+OFCs also significantly (*p* < 0.05) increased the pH of the rhizosphere soil, possibly because the interaction between strain TJ6 and iron minerals counteracts the acid solubility of the iron minerals and increases the pH of the rhizosphere soil. Compared with the control group, none of the passivating agents significantly (*p* < 0.05) affected the organic matter content of the rhizosphere soil (Figure 3b). In contrast to the CK group, Fh, OFCs, TJ6+Fh, and TJ6+OFCs significantly (*p* < 0.05) increased the Fe content in the rhizosphere soil (Figure 3c), indicating that these treatments increased the iron oxide content in the soil. Furthermore, the EC value of the rhizosphere soil rose significantly (*p* < 0.05) after the addition of Fh, likely due to its poor stability, making it prone to dissolution and re-release into the soil, thereby increasing the EC value. The EC value also increased in treatment groups with strain TJ6, possibly because the strain itself has the ability to activate and undergo redox reactions with iron ions, with free iron ions contributing to higher soil EC (Figure 3d). Additionally, strain TJ6 can activate iron oxides and release them into the soil system, and the urease secreted by TJ6 leads to an increase in soil pH. Strain TJ6 increased the content of free iron oxides in the soil, and the rise in free iron oxides also increased the soil EC value.

### 3.4. Contents of Total Organic Carbon and Dissoluble Organic Carbon in Rhizosphere Soil

**Figure 4:**
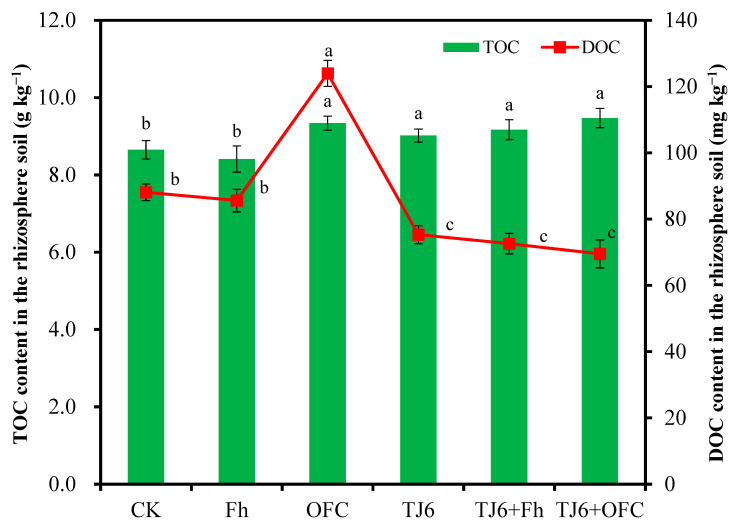
The effects of different treatments on the total organic carbon and dissoluble organic carbon in wheat rhizosphere soil. The error bars represent ± standard errors (n = 3). The green columns represent the content of TOC, and the red lines represent the content of DOC. The bars indicated by different letters are significantly different (*p* < 0.05) according to Tukey’s HSD test.

Compared to the CK group, Fh had no significant (*p* < 0.05) effect on the TOC content in rhizosphere soil, while OFCs, TJ6, TJ6+Fh, and TJ6+OFCs significantly (*p* < 0.05) increased the TOC content in rhizosphere soil (Figure 4). This was primarily because the OFC itself contains a certain amount of C, and strain TJ6 can secrete urease, which also enhances the C content in the soil. Furthermore, the addition of OFCs significantly (*p* < 0.05) increased the DOC content in rhizosphere soil, whereas the inoculation of strain TJ6 inhibited the release of DOC from OFCs (Figure 4), reducing C emissions and simultaneously strengthening the fixation of Cd by soil particles. Additionally, TJ6, TJ6+Fh, and TJ6+OFC significantly (*p* < 0.05) decreased (14.5–21.1%) the DOC content in rhizosphere soil, likely because strain TJ6 induced the conversion of DOC into fixed C, further reducing C emissions.

### 3.5. NH_4_^+^ and NO_3_^−^ Contents in the Rhizosphere Soil

**Figure 5:**
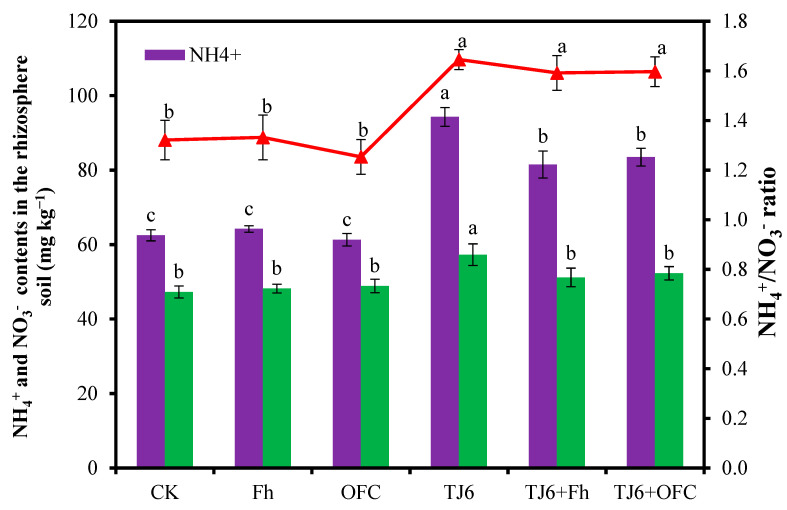
The effects of different treatments on the NH_4_^+^ and NO_3_^−^ contents and the NH_4_^+^/NO_3_^−^ ratio in wheat rhizosphere soil. The error bars represent ± standard errors (n = 3). Purple and green represent the contents of NH_4_^+^ and NO_3_^−^, respectively. The red line represents NH_4_^+^/NO_3_^−^ ratio. The bars indicated by different letters are significantly different (*p* < 0.05) according to Tukey’s HSD test.

The NH_4_^+^ and NO_3_^−^ contents in the rhizosphere soil of the CK group were 62.5 mg kg^−1^ and 47.3 mg kg^−1^, respectively (Figure 5). Inoculation with strain TJ6 significantly (*p* < 0.05) increased NH_4_^+^ content by 50.9% and NO_3_^−^ content by 21.1% in the rhizosphere soil compared to CK. In contrast, neither Fh nor OFCs showed significant (*p* < 0.05) effects on NH_4_^+^ or NO_3_^−^ contents. Furthermore, while the composite passivator had no significant (*p* < 0.05) impact on the NO_3_^−^ content, it markedly increased the NH_4_^+^ content by 30.4%. Notably, the NH_4_^+^/NO_3_^−^ ratio in TJ6-containing treatments was significantly (*p* < 0.05) higher than the ratios in the CK, OFC, and Fh treatments, suggesting a potential correlation between the NH_4_^+^/NO_3_^−^ ratio and Cd immobilization in rhizosphere soil.

### 3.6. Changes in Bacterial Community Structure in Wheat Rhizosphere Soil

**Figure 6:**
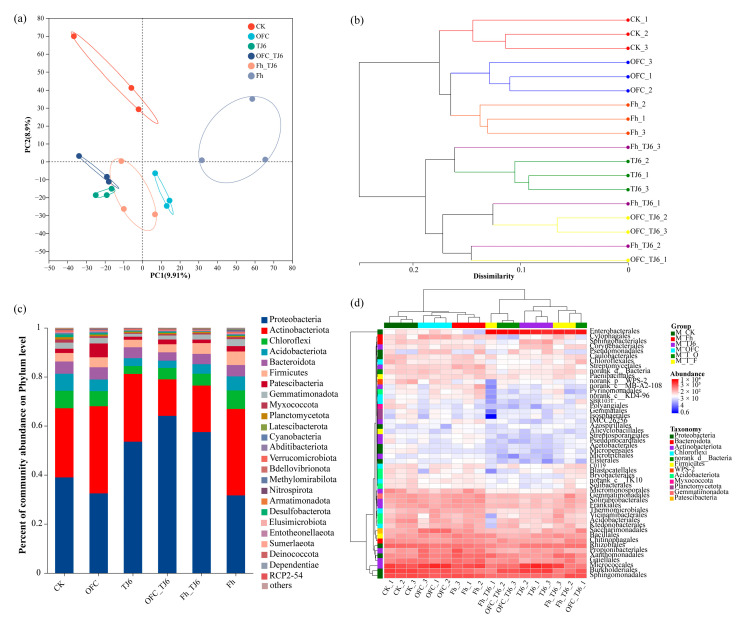
Analysis of rhizosphere soil–microbial community diversity and community structure. (**a**) PCoA analysis; (**b**) UPGMA tree; (**c**) analysis of microbial community composition at phylum level. (**d**) Analysis of microbial community composition at genus level.

Principal coordinate analysis (PCoA) showed three distinct clusters formed by the CK samples, Fh-treated samples, and OFC-treated samples, whereas the samples treated with TJ6, OFC+TJ6, and Fh+TJ6 were clustered together, indicating that inoculation with strain TJ6 significantly altered the structure of bacterial communities in the rhizosphere soil (Figure 6a). Similarly, the UPGMA tree showed that samples in the CK, Fh, and OFC treatments formed one large branch, while samples treated with TJ6, OFC+TJ6, and Fh+TJ6 formed another large branch, further indicating that strain TJ6 significantly affects the structure of bacterial communities in rhizosphere soil (Figure 6b). In all treatment groups, the proportions of Proteobacteria, Actinobacteriota, Chloroflexi, Acidobacteriota, Firmicutes, and Bacteroidota in the rhizosphere soil exceeded 90% (Figure 6c). Compared to the CK, OFC, and Fh treatments, TJ6, OFC+TJ6, and Fh+TJ6 significantly increased the relative abundances of Proteobacteria and Firmicutes, while they decreased those of Actinobacteriota and Chloroflexi. Additionally, compared to the CK, OFC, and Fh treatments, inoculation with TJ6, OFC+TJ6, and Fh+TJ6 increased the relative abundances of *Enterobacterales*, *Pseudomonadales*, *Gemmatimonadales*, *Sphingomonadales*, *Burkholderiales*, and *Rhizobiales* (Figure 6d), suggesting that these bacterial communities play an important role in resisting Cd stress.

### 3.7. Specific Bacterial Assemblages

**Figure 7:**
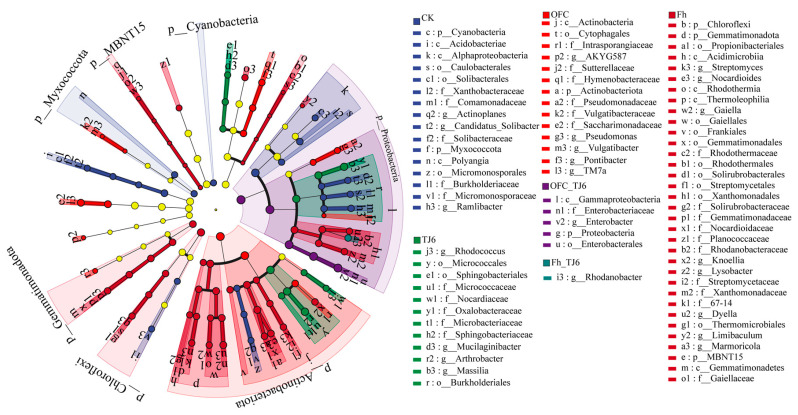
LEfSe analysis based on OTUs between four treatments. The cladogram shows the biomarker microbes of the microbial lineages from the domain to genus levels among the four different treatments.

In rhizosphere soil, certain key bacterial genera play a significant role in plant growth and development. LefSe analysis revealed that g__Actinoplanes, g__Ramlibacter, p__Cyanobacteria, and f__Micromonosporaceae were the key genera in the CK treatment (Figure 7). In contrast, TJ6, OFC+TJ6, and Fh+TJ6 treatments were characterized by key genera, such as p__Gemmatimonadota, p__Proteobacteria, g__Enterobacter, g__Rhodanobacter, g__Massilia, g__Arthrobacter, g__Nocardioides, and g__Gaiella. Literature reviews indicated that Proteobacteria and Gemmatimonadota exhibited tolerance in heavy-metal-contaminated environments, with their unique physiological traits enabling their survival and proliferation in polluted soils. They participated in nutrient cycles (e.g., carbon and nitrogen), improving soil conditions and creating a favorable environment for other microorganisms, thereby indirectly facilitating the transformation and immobilization of heavy metals [38,39,40]. Bacteria from the genera *Enterobacter* and *Rhodanobacter* reduced the bioavailability of heavy metals through biosorption, bioprecipitation, and redox reactions [19,41]. *Massilia* and *Nocardioides* secreted extracellular polymeric substances (EPSs) and siderophores, enhancing the immobilization of heavy metals [42,43]. *Arthrobacter*, commonly found in contaminated soils, can immobilize heavy metals via biosorption and biomineralization [44]. In summary, the Fh+TJ6 and OFC+TJ6 treatments synergistically enhanced Cd immobilization and wheat growth by enriching the rhizosphere soil with bacterial genera capable of producing EPSs, inducing phosphate precipitation, facilitating biosorption, and promoting nutrient cycling.

### 3.8. Prediction of Rhizosphere Bacterial Community Functions

**Figure 8:**
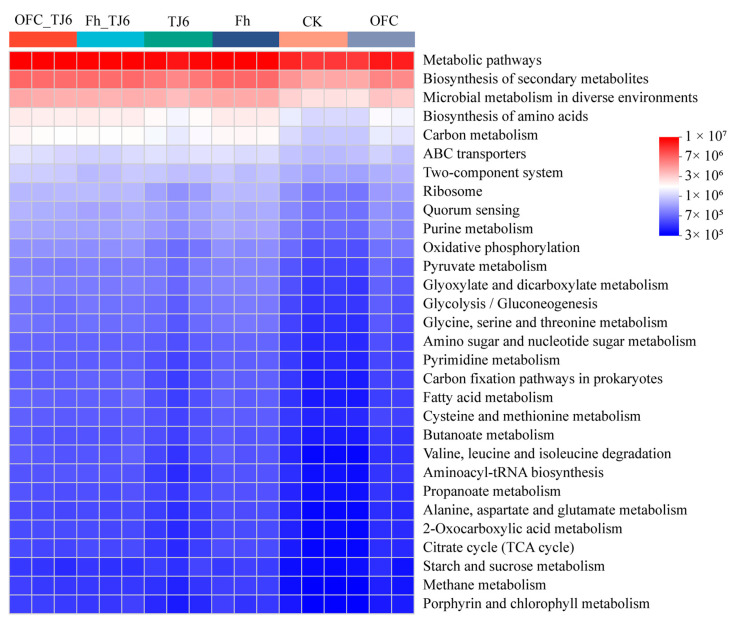
PICRUSt2 function prediction. The horizontal axis represents the sample name, and the vertical axis represents the pathway level 3 function name. The color gradient of the color blocks is used to display changes in the abundance of different functions in the sample. The legend represents the numerical values represented by the color gradient.

PICRUSt2 function prediction is used to infer information about the functions of microbial communities in environmental samples. By analyzing the functional composition and abundance, PICRUSt2 provides insights into potential microbial functional characteristics during environmental changes. Compared to the CK group, the groups inoculated with strain TJ6 (TJ6, OFC+TJ6, and Fh+TJ6) exhibited significantly increased abundances of genes related to carbon metabolism, the biosynthesis of amino acids, ABC transporters, oxidative phosphorylation, and quorum sensing in the rhizosphere soil (Figure 8). Notably, in the composite passivator treatments, the abundances of genes associated with carbon metabolism, the biosynthesis of amino acids, and oxidative phosphorylation were markedly enhanced, suggesting that bacterial communities carrying these functions extensively colonized the wheat rhizosphere soil.

### 3.9. Correlation Analysis

**Figure 9:**
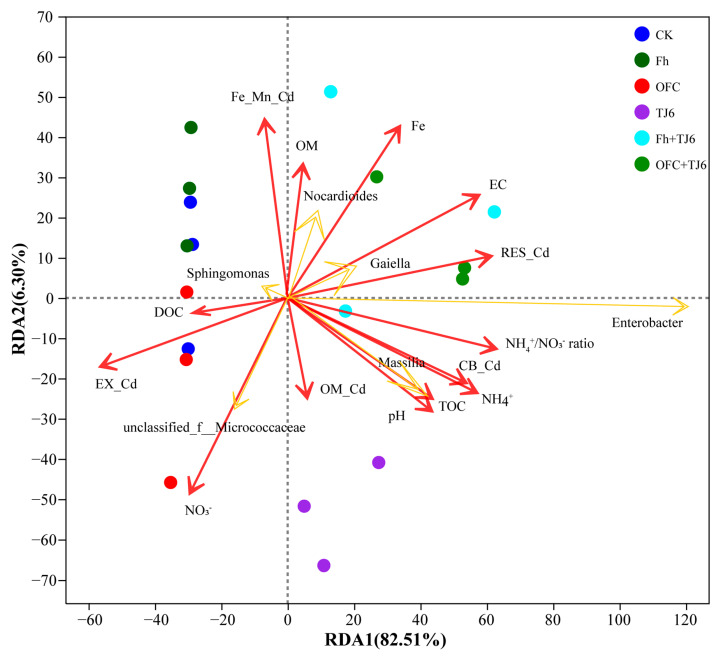
RDA of the exchangeable Cd and soil environmental variables in the rhizosphere soil of wheat.

The overall RDA model was statistically significant (F = 3.84, *p* < 0.05), indicating that the selected environmental variables collectively explained a significant proportion of the variation in bacterial community composition. The constrained variance explained by the model was 22.3%. Permutation tests (999 permutations) further showed that the first constrained axis (RDA1) was significant (F = 4.2, *p* < 0.05) and explained 14.1% of the variance, whereas the second constrained axis (RDA2) was not significant (*p* = 0.18). The RDA results indicate that the EX-Cd content in the wheat rhizosphere soil was significantly negatively correlated (r = −0.78, *p* < 0.05) with pH, EC content, free Fe content, TOC content, and the NH_4_^+^/NO_3_^−^ ratio (Figure 9). Furthermore, the relative abundances of *Enterobacter*, *Nocardioides*, *Gaiella*, and *Massilia* in the rhizosphere soil were also negatively correlated (r = −0.64, *p* < 0.05) with EX-Cd levels (Figure 9). In summary, the application of passivators (strain TJ6, Fh, and OFCs) in rhizosphere soil increased the soil pH, EC content, free Fe content, TOC content, and NH_4_^+^/NO_3_^−^ ratio, thereby reducing the bioavailability of Cd. Concurrently, the relative abundances of *Enterobacter*, *Nocardioides*, *Gaiella*, and *Massilia* were significantly enhanced, synergistically contributing to Cd immobilization and reducing Cd uptake by wheat.

## 4. Discussion

Soil Cd pollution poses a severe threat to ecological environments, agricultural production, and human health, necessitating the urgent exploration of effective remediation strategies [45]. The synergistic interactions among soil bacteria, organic carbon, and iron oxides in Cd adsorption and immobilization provide novel insights and directions for addressing this issue [46]. In this study, the combined application of the urease-producing bacterial strain TJ6 and Fh/OFCs effectively immobilized Cd in the rhizosphere soil and reduced Cd accumulation in wheat grains. The underlying mechanisms include the following: (1) significant increases in NH_4_^+^-N content and the NH_4_^+^-N/NO_3_^−^-N ratio in the rhizosphere soil elevated soil pH, thereby reducing Cd mobility (Figure 5). (2) Enhanced DOC binding with soil particles, alongside the increased iron oxide content and EC of rhizosphere soil, strengthened the Cd retention capacity (Figure 3 and Figure 4). (3) The enrichment of functional bacterial genera in the rhizosphere soil—including *Enterobacter*, *Rhodanobacter*, *Massilia*, *Nocardioides*, and *Arthrobacter*, which exhibit capabilities to produce extracellular polymeric substances (EPSs), induce phosphate precipitation, facilitate biosorption, and promote C/N cycling—synergistically enhanced Cd immobilization and wheat growth (Figure 7). These findings highlight the potential of microbial–organic–mineral collaborative strategies for mitigating Cd contamination in agricultural systems.

The synergistic interaction between the urease-producing bacterial strain TJ6 and Fh/OFCs establishes multiple Cd immobilization barriers by reshaping rhizospheric nitrogen transformation dynamics and organic carbon metabolism. Urease (EC 3.5.1.5) secreted by strain TJ6 catalyzed urea hydrolysis: CO(NH_2_)_2_ + H_2_O → 2NH_3_ + CO_2_. The resulting NH_3_ and CO_2_ were converted to NH_4_^+^ and CO_3_^2−^, elevating rhizosphere pH from 7.2 to 7.4. This triggered the reaction Cd^2+^ + CO_3_^2−^ → CdCO_3_**↓**. Simultaneously, pH-induced deprotonation of FeOOH surface hydroxyl groups (≡Fe-OH) generated more ≡Fe-O^−^ sites, enhancing the specific adsorption (inner-sphere complexation) of Cd^2+^. Strain TJ6 further oxidized FeO to Fe_2_O_3_ via cytochrome c oxidase, where Fe^2+^/Fe^3+^ redox cycling facilitated Cd coprecipitation as Fh-Cd-CO_3_ ternary complexes [47]. Urease-mediated urea hydrolysis significantly elevates NH_4_^+^ concentrations, which suppresses nitrifying bacterial activity [48], reduces nitrification intensity, and increases the NH_4_^+^/NO_3_^−^ ratio (from 1.32 to 1.59 in our study). This nitrogen speciation shift raises the rhizosphere pH by 0.3–0.8 units through reduced H^+^ production, promoting Cd^2+^ conversion into low-solubility phases (e.g., CdCO_3_) while enhancing competitive inhibition at plant uptake sites [49], where NH_4_^+^ occupancy reduces Cd^2+^ influx due to the higher affinity of the Nramp5 transporter for Cd^2+^ (*Km* ≈ 1.2 μM) over NH_4_^+^ (*Km* ≈ 50 μM) [50,51]. Concurrently, the OFC enhances Cd immobilization via Fh’s large specific surface area (200–400 m^2^ g^−1^) and Fe-OH, achieving a Cd^2+^ monolayer adsorption capacity of 45.2 mg g^−1^ [52,53]. Yang et al. [54] reported that bioavailable Cd in the soil decreased by 65.2% after treatment with the urease-producing bacterial Y15 strain. In this study, the strain TJ6-OFC consortium reduced wheat grain Cd content from 0.31 mg kg^−1^ to 0.08 mg kg^−1^, which is below China’s food safety threshold (0.1 mg kg^−1^), demonstrating dual benefits, namely, environmental Cd immobilization and agricultural product safety.

Iron oxides (e.g., ferrihydrite, hematite, and magnetite), naturally occurring heavy metal adsorbents in soil, exhibit a strong affinity for Cd. These oxides immobilize Cd through mechanisms such as surface complexation, coprecipitation, and ion exchange [55,56]. Under oxidizing conditions, iron (hydr)oxide precipitates are formed and effectively encapsulate and stabilize Cd [51]. As variable-charge minerals, iron oxides are highly sensitive to pH during interactions with heavy metals. Generally, increased pH promotes deprotonation of iron oxide surfaces, reducing their positive charge density and enhancing the adsorption of positively charged heavy metal cations. For instance, Du et al. [57] demonstrated that the Fh adsorption capacity for Pb(II) increased from ~25% to 100% as the pH rose from 4.0 to 5.5. Organic carbon materials (e.g., biochar and humic acid), rich in oxygen-containing functional groups such as -COOH, -OH, and C=O, can form composite binding sites with Fe-OH on iron oxide surfaces, enhancing heavy metal complexation [58,59]. Compared to pure Fh, OFCs exhibit variable adsorption capacities for heavy metals, depending on the underlying mechanisms. For example, OFCs formed with humic/fulvic acids significantly improve Pb and Cd adsorption at neutral to low pH compared to ferrihydrite alone [60]. This enhancement arises from the limited intrinsic adsorption capacity of Fh in this pH range, while humic/fulvic acids provide additional organic binding sites. Conversely, some studies report reduced adsorption on OFCs due to site occupation or masking caused by Fe(III)–organic interactions [27]. In this study, corn-straw-derived OFCs exhibited weaker Cd immobilization than Fh, likely due to adsorption site blockage by organic loading. Similarly, Wang et al. [61] observed a decline in effective Ni adsorption sites in OFCs. In the ternary bacteria–organic carbon–iron oxide system, microbial extracellular polymeric substances (EPSs) and biofilms synergistically coat iron oxides with organic carbon, forming a “biomineral” composite layer that provides additional adsorption sites [62]. Cd^2+^ adsorbed by bacteria can be further encapsulated by adjacent iron oxides, forming stable ternary “bacteria–Cd–iron oxide” complexes [28]. In this study, the urease-producing bacteria TJ6 enhanced Cd adsorption and immobilization by OFCs in rhizosphere soil, reducing Cd mobility and subsequent Cd accumulation in wheat grains. Microbial metabolism altered local rhizosphere pH and Eh, facilitating Cd^2+^ coprecipitation with Fe^3+^ into stable minerals. The TJ6+OFC treatment significantly increased rhizosphere soil pH and EC, further enhancing Cd retention. Thus, the ternary system establishes a hierarchical Cd adsorption interface: primary layer: Cd^2+^ complexation by organic carbon functional groups (-COOH); secondary layer: Cd^2+^ coprecipitation with iron oxide surface hydroxyls (Fe-OH); and tertiary layer: additional binding sites on bacterial EPSs and cell walls. This multilayered strategy highlights the synergistic potential of microbial–organic–mineral interactions for sustainable Cd remediation in contaminated soils.

In this study, the addition of passivators significantly reshaped the rhizobacterial community structure of wheat, as revealed by high-throughput sequencing. The relative abundances of Proteobacteria and Gemmatimonadota increased by 1.8- to 2.5-fold and 1.2- to 1.6-fold, respectively, alongside the marked enrichment of key functional genera, such as *Enterobacter*, *Rhodanobacter*, *Massilia*, *Arthrobacter*, *Nocardioides*, and *Gaiella*. This directional community succession synergistically reduced Cd bioavailability through multipath mechanisms. Biosorption and surface complexation were enhanced by Proteobacteria, which adsorb Cd via cell surface charges or specific structures, accumulating ions intra- or extracellularly to mitigate toxicity [37,63,64]. Notably, *Enterobacter* sp. TJ6, with abundant -NH_2_ and -COOH groups, exhibited a negative surface charge under rhizosphere microalkaline conditions (pH 7.2–7.8), achieving a Cd adsorption capacity of 0.32 mg g^−1^ per cell via electrostatic interactions [29]. Element-metabolism-driven biomineralization was facilitated by Gemmatimonadota, which enhanced nutrient cycling (C, N, S, Fe) to improve soil conditions, indirectly promoting Cd immobilization [65]. *Desulfovibrio* facilitates the reduction of SO_4_^2−^, thus promoting CdS precipitation [66]. *Massilia* spp. secreted EPSs and siderophores to stabilize heavy metals [42,43], while *Arthrobacter*, commonly found in contaminated soils, can immobilize heavy metals via biosorption and biomineralization [44]. Organic matter transformation and competitive inhibition further contributed, as soil organic ligands complexed Cd, reducing its mobility [67]. Collectively, these interactions established a three-tier Cd immobilization network: (1) short-term rapid fixation via surface adsorption and extracellular complexation; (2) medium-term stabilization through Fe/N/C-driven mineralization; and (3) long-term bioavailability reduction via organic matter transformation and competitive inhibition. These findings provide a theoretical foundation for microbial–chemical combined remediation strategies in Cd-contaminated soils.

In our research, the combined application of strain TJ6 and Fh/OFCs in the rhizosphere soil reduced the Cd content in wheat grains by approximately 77.4%. The application of chemical immobilizers such as hydroxyapatite or nano-zinc oxide decreased the Cd content in wheat grains by about 44.5% to 63.9% [68,69], while *Ralstonia eutropha* Q2-8 and *Exiguobacterium aurantiacum* Q3-11 could reduce the Cd content in wheat grains by approximately 12% to 32% [70]. This indicates that the combined application of strain TJ6 and Fh/OFCs has a strong ability to control the absorption of Cd by wheat and demonstrates unique advantages. Although we made efforts to control the experimental conditions, potential confounding variables may have existed in the pot experiments. We have minimized these effects as much as possible through a randomized design, sufficient repetitions, and standardized management and exercised caution in the interpretation of the results. Moreover, the observed data variability in this study primarily stems from the following sources: inherent biological variability, microbial community heterogeneity, spatial variability in soil sampling, and analytical error. Despite this variability, key indicators (e.g., increased soil pH, significantly reduced available Cd, decreased Cd accumulation in grains, and enrichment of specific functional bacterial genera) in the TJ6+Fh/OFC treatment group consistently showed statistically significant (*p* < 0.05) and biologically meaningful trends compared to the control. This robustly supports our core conclusion regarding the effectiveness of the combined strategy.

This study employed a pot experiment, which provided an advantage for precisely studying the mechanism and short-term effects of specific microbial–mineral associations under controlled conditions. However, field conditions are relatively complex, and the Cd fixation efficiency and the reduction in Cd absorption on wheat observed in this study may change when applied to actual field environments. Further research involving long-term field experiments in typical polluted farmland is urgently needed to verify the practical application effects, economic feasibility, and environmental safety of the amendments used in this study. The sample size in this study was relatively small, with only three replicates for each treatment. The experimental results thus have a certain degree of randomness and there is a possibility of batch variation. Therefore, in subsequent studies, more replicates should be set up to enhance the reliability of the results. At the same time, the combined analysis of soil metabolomics and microbial communities should be strengthened to explore the corresponding mechanisms at the metabolite-species level. The study period covered a complete wheat growing season. Although this is sufficient to observe significant Cd fixation effects and reduced Cd absorption in grains, the long-term stability and sustainability are not yet clear. Long-term site-specific experiments will help verify the functional stability of the strain. Additionally, the soil used in this experiment was weakly alkaline loam (pH 7.2), and a weakly alkaline environment is conducive to the stability of iron oxides and the occurrence of certain Cd fixation mechanisms (such as precipitation or specific adsorption). However, the effectiveness of this strategy may vary in soils with different physical and chemical properties (such as acidic red soil, sandy soil, or soils with significant differences in organic matter content).

## 5. Conclusions

In this study, the combined application of the urease-producing bacteria TJ6 with Fh/OFCs effectively reduced the bioavailable Cd content in rhizosphere soil and minimized Cd accumulation in wheat grains. The remediation mechanism involved the multidimensional regulation of the rhizosphere microenvironment: (1) elevated soil pH, enhanced iron oxide content, and increased EC collectively strengthened Cd adsorption and immobilization on soil particles; (2) the increased NH_4_^+^ concentration and NH_4_^+^/NO_3_^−^ ratio suppressed Cd uptake by wheat roots through competitive inhibition at ion transport sites while further alkalinizing the rhizosphere; (3) reduced DOC content indicated enhanced carbon sequestration and Cd fixation via organic–inorganic interactions, likely driven by the formation of stable Fe-O-Cd-O-C ternary complexes; (4) restructured bacterial communities, particularly the enrichment of Proteobacteria, Gemmatimonadota, and functional genera (*Enterobacter*, *Rhodanobacter*, *Massilia*, *Nocardioides*, and *Arthrobacter*), synergistically immobilized Cd through biosorption, biomineralization, and metabolic reprogramming. While these findings highlight the potential of bacteria–organic–mineral interfacial synergism for Cd passivation, critical knowledge gaps remain regarding the long-term stability of ternary complexes, field-scale applicability under varying soil conditions, and molecular-scale mechanisms governing component interactions. Addressing these challenges will be essential for translating laboratory-scale success into practical, sustainable remediation strategies.

## Data Availability

The original contributions presented in this study are included in the article. Further inquiries can be directed to the corresponding authors.
